# Hybrid Membranes Based on Track-Etched Membranes and Nanofiber Layer for Water–Oil Separation and Membrane Distillation of Low-Level Liquid Radioactive Wastes and Salt Solutions

**DOI:** 10.3390/membranes15070202

**Published:** 2025-07-04

**Authors:** Arman B. Yeszhanov, Aigerim Kh. Shakayeva, Maxim V. Zdorovets, Daryn B. Borgekov, Artem L. Kozlovskiy, Pavel V. Kharkin, Dmitriy A. Zheltov, Marina V. Krasnopyorova, Olgun Güven, Ilya V. Korolkov

**Affiliations:** 1 The Institute of Nuclear Physics, Ibragimov str. 1, Almaty 050032, Kazakhstan; arman_e7@mail.ru (A.B.Y.); a.shakayeva@inp.kz (A.K.S.); mzdorovets@inp.kz (M.V.Z.);; 2 Department of Chemistry, Hacettepe University, Beytepe, Ankara 06800, Turkey; guven@hacettepe.edu.tr

**Keywords:** track-etched membranes, nanofibers, hybrid membranes, water–oil emulsions separation, membrane distillation, desalination, radioactive wastes

## Abstract

In this work, hybrid membranes were fabricated by depositing polyvinyl chloride (PVC) fibers onto PET track-etched membranes (TeMs) using the electrospinning technique. The resulting structures exhibited enhanced hydrophobicity, with contact angles reaching 155°, making them suitable for applications in both water–oil mixture separation and membrane distillation processes involving low-level liquid radioactive waste (LLLRW), saline solutions, and natural water sources. The use of hybrids of TeMs and nanofiber membranes has significantly increased productivity compared to TeMs only, while maintaining a high degree of purification. Permeate obtained after MD of LLLRW and river water was analyzed by conductometry and the atomic emission spectroscopy (for Sr, Cs, Al, Mo, Co, Sb, Ca, Fe, Mg, K, and Na). The activity of radioisotopes (for ^124^Sb, ^65^Zn, ^60^Co, ^57^Co, ^137^Cs, and ^134^Cs) was evaluated by gamma-ray spectroscopy. In most cases, the degree of rejection was between 95 and 100% with a water flux of up to 17.3 kg/m^2^·h. These membranes were also tested in the separation of cetane–water emulsion with productivity up to 47.3 L/m^2^·min at vacuum pressure of 700 mbar and 15.2 L/m^2^·min at vacuum pressure of 900 mbar.

## 1. Introduction

Membrane technologies cover increasingly large areas of application for water purification from salts, separation of water–oil emulsions, and various chemical industry applications. There is also an increasing number of publications on the use of membrane technologies for radionuclide removal [1,2,3,4,5,6,7,8] because of the growth in nuclear energy production due to the high global demand for energy. Despite its reliability and stability, nuclear energy production is associated with significant issues, primarily the formation of various byproducts from nuclear reactions. Low-level liquid radioactive waste (LLLRW) may originate from various sources, including radioisotope production for medical purposes, routine operations at nuclear facilities, activities at nuclear industry sites, and incidents involving anthropogenic radiation releases [1,2,3,4,5,9].

LLLRW can cause irreparable harm to the environment by contaminating water bodies, such as groundwater, rivers, and oceans, which can negatively impact human health. When radionuclides enter the human body, they can lead to various cancers and even death. Therefore, the issue of purifying water from radionuclides is crucial and important not only for the nuclear industry, but also globally [6,7,8,10].

Today, various methods are used to address the challenges of cleaning liquid radioactive waste, including thermal [11,12,13], sorption [14,15,16], and membrane techniques [17,18,19,20]. Thermal methods, such as evaporation and drying, are effective but require large amounts of heat, which is a significant drawback. Sorption purification methods, such as adsorption and ion exchange, are also commonly used. However, membrane processes are considered the most promising for the purification of LLLRW. Techniques like ultrafiltration (UF), microfiltration (MF), nanofiltration (NF), forward osmosis (FO), and reverse osmosis (RO) are often employed [21,22,23,24,25,26,27,28,29,30]. Over the past decade, interest in membrane distillation (MD) has grown due to its high degree of decontamination and rejection. Unlike other separation methods, MD offers several advantages: it does not require excess heat, achieves a high degree of purification for non-volatile components, and operates at low pressures [19,20,31,32,33,34,35].

MD is a thermally driven separation process that exploits the difference in vapor pressure across a hydrophobic, microporous membrane. One side of the membrane is in contact with a heated aqueous feed solution, while the other side is maintained at a lower temperature (depending on the MD configuration). The temperature difference across the membrane induces a vapor pressure gradient, which acts as the driving force for water vapor to move through the membrane pores. Owing to the hydrophobic characteristics of the membrane, it selectively allows only the vapor phase to pass, effectively preventing liquid water from entering the pores. Consequently, substances with low volatility, such as salts, heavy metals, and radioactive elements, remain in the feed solution [36,37].

Membrane distillation (MD) can be implemented in various designs, including direct contact (DCMD), air gap (AGMD), vacuum (VMD), and sweeping gas (SGMD) configurations [38,39,40,41,42]. Among these, DCMD is the most widely used due to its simplicity and compact design, typically achieving water fluxes of 10–25 kg/m^2^·h at feed temperatures of 60–80 °C. AGMD and VMD, while more complex, can offer higher fluxes (up to 30–40 kg/m^2^·h) and improved energy efficiency due to reduced heat loss and enhanced vapor transfer, respectively. SGMD is less common but can be advantageous in certain industrial applications requiring continuous gas sweeping and lower heat conductivity across the membrane.

In membrane distillation, hydrophobic polymer-based membranes are typically employed. Materials such as poly(vinylidene fluoride) (PVDF), polytetrafluoroethylene (PTFE), polypropylene (PP), and polyethylene (PE) are widely used, offering contact angles in the range of 110° to 140° and porosity values generally between 60% and 80% [31,43,44]. These membranes provide high thermal and chemical stability, but their relatively thick structures (typically 100–200 μm) can limit vapor flux and increase thermal resistance.

Track-etched membranes (TeMs) based on poly(ethylene terephthalate) (PET) have also been studied for MD applications, especially in the context of LLLRW purification [19,20]. PET TeMs are characterized by precisely defined cylindrical pores (with diameters ranging from 100 nm to over 2 μm), narrow pore size distribution, high pore density (10^6^–10^9^ pores/cm^2^), and small thickness (6–23 μm), which together provide excellent size-selectivity and low mass transfer resistance [45,46,47,48,49,50,51]. However, their native hydrophilicity and low surface roughness often require surface modification to enable stable vapor-phase transport in MD.

Contaminant buildup on the membrane can lead to pore wetting and a decline in permeate flux. Additionally, PET TeMs have insufficient hydrophobic characteristics and need modification for effective use in MD. Various methods for hydrophobizing PET TeMs have been previously investigated, including UV-graft polymerization, covalent bonding of fluoro- and chlorosilanes, and the immobilization of silica nanoparticles on the surface [19,20,52,53,54]. To further increase hydrophobicity and reduce pore wetting, the concept of hybrid membranes, which incorporate both hydrophilic and hydrophobic components, has been investigated [55,56].

There are several methods for fabricating hydrophobic membranes, including chemical vapor deposition, electrospray, phase inversion, sol–gel method, and electrospinning [57,58,59]. Among these, electrospinning has been widely recognized as an effective technique for membrane production and surface modification due to its versatility and scalability. Electrospun membranes typically exhibit high porosity (up to 80–90%), small and tunable fiber diameters ranging from 100 nm to 2 μm, and large surface area-to-volume ratios, which contribute to improved vapor transport and enhanced hydrophobicity (contact angles often exceeding 140°) [58,59]. In membrane distillation (MD), nanofibrous membranes produced via electrospinning have demonstrated high salt rejection (>99.9%) and water fluxes ranging from 1.83 to 78 kg/m^2^·h, depending on feed composition and membrane architecture [4,5,36]. Previously published studies on the treatment of LLLRW using modified PET TeMs have reported decontamination factors above 1700 for ^137^Cs and more than 85 for ^60^Co [19,20]. However, the relatively low intrinsic permeability of PET-based TeMs—typically below 2 kg/m^2^·h—necessitates the use of electrospun coatings or hybrid structures to enhance water flux without compromising selectivity.

This work presents, for the first time, the results of obtaining hybrid PET TeMs with poly(vinyl chloride) (PVC) fibers on their surfaces using the electrospinning method for the purification of model salt solutions; in addition, real water purification was also performed for river water, as well as LLLRW. The obtained hydrophobic hybrid membranes with a high water contact angle (CA) were also tested in the separation of water–oil emulsions. Low water fluxes of TeMs were overcome by increasing the pore diameter of TeMs in the range of 1159 ± 27–2494 ± 81 nm, with the prevention of hydrophobic properties (up to 155°) using PVC fibers.

## 2. Materials and Methods

### 2.1. Materials

PET film was obtained from Mitsubishi Polyester Film (Wiesbaden, Germany). High molecular weight poly(vinyl chloride) (PVC) (Sigma-Aldrich, Zwolle, The Netherlands), along with tetrahydrofuran (THF) (Sigma-Aldrich, St. Louis, MO, USA) and dimethylformamide (DMF) (Sigma-Aldrich, Darmstadt, Germany), were used in the experiments. Analytical grade sodium chloride was used in the experiments, and all solutions were prepared using deionized water with a resistivity of 18.2 MΩ·cm.

### 2.2. Preparation of TeMs

PET track-etched membranes (TeMs) were fabricated by irradiation PET films with krypton ions at an average energy of 1.75 MeV per nucleon and a fluence of 1 × 10^6^ ions/cm^2^ using the DC-60 cyclotron (Astana, Kazakhstan). Subsequent chemical etching was performed in a 2.2 M NaOH solution at 85 °C. Upon completion of etching, the membranes were rinsed with acetic acid, followed by deionized water, and then air-dried and stored at ambient conditions.

### 2.3. Preparation of Hybrid Membranes by Electrospinning

Preparation of hybrid membranes was carried out by applying PVC nanofibers by electrospinning using a tabletop installation for electrospinning CY-ES30K-H (Zhengzhou CY Scientific Instrument Co, Zhengzhou, China). First, a polymer solution was prepared in tetrahydrofuran and N,N-dimethylformamide with a 1:1 (*v/v*) ratio with various concentrations between 10–17%. Electrospinning parameters: voltage (from 8 kV to 15 kV), the feed rate of PVC solutions (from 0.3 to 1 mL/h), and the distance between the needle and the collector remained constant at 15 cm.

### 2.4. Methods of Characterization of the Membrane

Fourier-transform infrared (FTIR) spectra were obtained using an InfraLUM FT-08 spectrometer (Lumex, Saint Petersburg, Russia) equipped with an ATR accessory (PIKE, Madison, WI, USA), operating in the 400–4000 cm^−1^ range with 25 scans and a resolution of 2 cm^−1^. The pore diameter of the TeMs was determined by the gas flow rate technique under a pressure of 20 kPa.

The surface morphology and elemental composition of the TeMs were examined using a Hitachi TM 3030 scanning electron microscope coupled with a Bruker XFlash MIN SVE EDX system (Bremen, Germany), operated at an accelerating voltage of 15 kV. Morphological and compositional data were averaged over three independent measurements.

Contact angle (CA) measurements were conducted at room temperature by the static drop method, using a digital microscope with 1000× magnification. A 15 µL droplet was placed at five distinct locations on each sample to ensure reproducibility.

Thermogravimetric analysis (TGA) was performed using a Themys ONE analyzer (Setaram, Caluire, France) over a temperature range of 0 to 800 °C under an argon atmosphere, with a constant heating rate of 10 °C/min.

The interfacial adhesion strength between electrospun PVC nanofiber layers and PET TeMs was quantitatively assessed using a progressive load scratch test on the equipment UNITEST-750 (Paltro, Bangalore, India). A spherical indenter was used to apply increasing normal force across a 10 mm stroke length. The effective contact width was 1 mm, resulting in a contact area of A = 1 × 10^−5^ m^2^. The maximum tractional force prior to delamination was considered as the critical force F_cr_, and adhesion strength was calculated as: δ_adh_ = F_cr_/A. Five measurements were performed. The applied normal load increased from 2 to 6–7 N at a constant loading rate of 0.1 N/s.

### 2.5. Testing of Hybrid Membranes in the Separation of Water–Oil Emulsions

Membrane samples with a surface area of 0.001256 m^2^ were tested under reduced pressure conditions ranging from 900 to 700 mbar using a VACSTAR Control vacuum pump (IKA, Staufen im Breisgau, Germany). Filtration experiments were carried out using a setup previously described in [60]. Water–cetane emulsions were prepared at a volume ratio of 1:100 by high-speed mixing with an Ultra-Turrax T18 disperser (IKA, Staufen im Breisgau, Germany) operating at 24,000 rpm for 1 min.

### 2.6. Membrane Distillation

Membrane distillation experiments were carried out in a direct contact membrane distillation (DCMD) setup, the configuration of which has been described in detail in previous studies [61,62]. Permeate flux was determined gravimetrically. The feed solutions included sodium chloride solutions with concentrations ranging from 7.5 to 30 g/L, as well as samples of low-level liquid radioactive waste (LLLRW) sourced from the WWR-K research reactor (Almaty, Kazakhstan). Salt rejection efficiency was assessed based on conductivity measurements using a Hanna Instruments HI2030-01 conductometer (Woonsocket, RI, USA).

To analyze the elemental composition of the feed and permeate solutions in the cases of LLLRW and river water, an OPTIMA-8000 ICP-OES system (PerkinElmer, Waltham, MA, USA) (spectral range 165–900 nm; resolution < 0.009 nm at 200 nm) was employed. Gamma-ray spectroscopy was performed using a Canberra GM1520 system (Canberra Industries, Meriden, CT, USA) equipped with a germanium semiconductor detector, covering an energy range of 25–3000 keV, to quantify the activity of selected radionuclides.

## 3. Results and Discussion

### 3.1. Preparation and Characterization of Membranes

**Figure 1:**
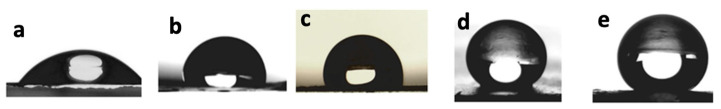
CA of pristine PET TeMs (**a**), PET TeMs-PVC obtained with concentration of PVC of 10% (**b**), 12% (**c**), 15% (**d**), 17% (**e**).

Hybrid membranes with hydrophobic properties were obtained by PVC fiber deposition on PET TeMs from both sides using the electrospinning method. A mixture of THF and DMF with a 1:1 ratio (by volume) is most often used as a PVC solvent for electrospinning application (other solvent ratios, as well as DMA, pure DMF, and THF, can also be used) [63]. The voltage and feed rate of the polymer solution were optimized to obtain polymer fibers: 0.5 mL/h and 12 kV; the distance from the needle tip to the collimator was kept constant at 15 cm. The effect of PVC concentration (10–17%) on the hydrophobic properties of the resulting membranes was investigated by measuring CA, since the CA is one of the key parameters that affects the success of the use of membranes in the separation of water–oil emulsions, especially in MD. The photographs of water droplets are presented in Figure 1.

With increasing PVC concentration, hydrophobicity increases. CA of initial PET TeMs is 55°, as well as for PET TeMs-PVC; 104°, 105°, 145°, 155° were obtained with the concentrations of 10%, 12%, 15%, 17%, respectively. The CA of PVC fiber obtained under different conditions can be 135–152° [64,65], which is comparable to the obtained results.

The thickness of the obtained membrane was controlled using an electronic thickness gauge. The thickness of the TeMs obtained by applying PVC on both sides of PET TeMs did not significantly change with the polymer concentration and amounted to 35 ± 7 µm, that is, considering that the thickness of the initial PET TeMs is 10 ± 1 µm, each side of the membrane was applied with a layer of PVC fibers as thick as 12.5 µm. The thickness of the layer was controlled by the time of fiber application. The surface density of PVC nanofibers deposited on the PET TeMs was also controlled and maintained at an average value of 2.58 ± 0.48 mg/cm^2^.

**Figure 2:**
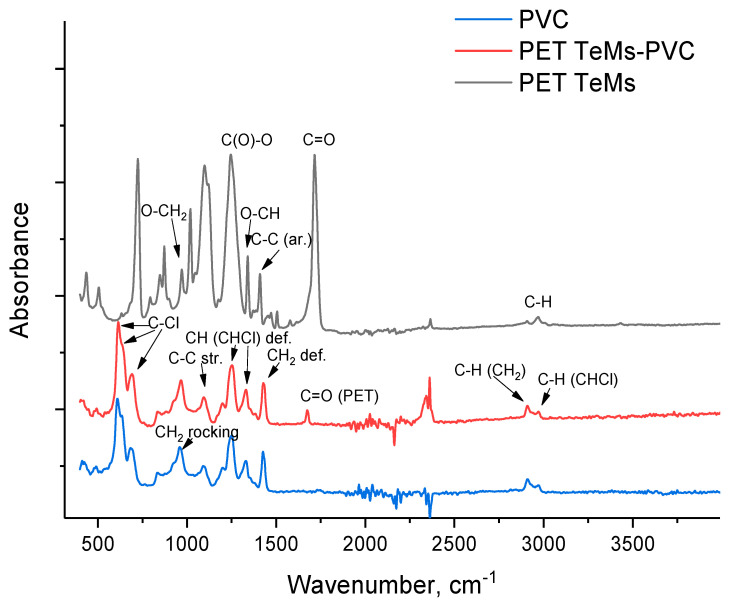
FTIR spectra of original PET TeMs (black line), PET TeMs-PVC (red line), and pure PVC (blue line).

To determine the functional groups before and after modification, FTIR spectra were recorded (Figure 2). The original PET TeMs shows characteristic absorption bands at 2970 cm^−1^ (benzene ring, C-H), 1410, 1430, 1616, 1470 cm^−1^ (aromatic vibrations of the carbon skeleton), 2912 cm^−1^ (aliphatic C-H), 1713 cm^−1^ (C=O), 970 cm^−1^ (O-CH_2_), 1340 cm^−1^ (O-CH), and 1238 cm^−1^ (C(O)-O). Electrospinning of PVC nanofibers on PET TeMs leads to the appearance of peaks characteristic of PVC only (2972, 2909, 1675, 1430, 1333, 1255, 1198, 1092, 966, 692, 610 cm^−1^) [66], and only peak at 1715 cm^−1^ related to PET are presented because the spectra were taken from the surface using an ATR attachment, and PVC fibers covered the surface of the membrane and other PET peaks are overlapped by PVC.

**Figure 3:**
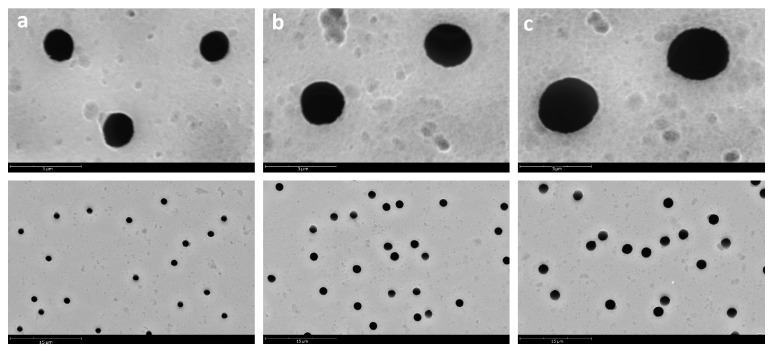
SEM microphotographs of PET TeMs with pore diameters of 1159 ± 27 nm (**a**), 1727 ± 73 nm (**b**), and 2494 ± 81 nm (**c**).

In this way, PVC fibers were electrospun onto PET TeMs with three different pore diameters (1159 ± 27, 1727 ± 73, and 2494 ± 81 nm). SEM images of the surface of the original PET TeMs were presented in Figure 3.

**Figure 4:**
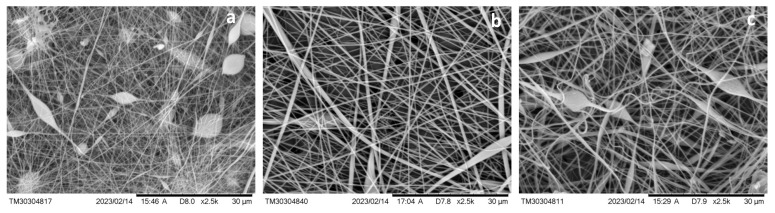
SEM microphotographs of PET TeMs covered with PVC fibers obtained with PVC concentration of 12% (**a**), 15% (**b**), and 17% (**c**).

Figure 4 presents SEM images of the hybrid membranes, clearly illustrating the formation of electrospun fibers on the surface of the PET track-etched membranes (PET TeMs). The fiber morphology varies depending on the concentration of polyvinyl chloride (PVC) used in the electrospinning solution. At a PVC concentration of 12%, relatively fine fibers with an average diameter of approximately 0.25 μm were formed. Increasing concentration to 15% resulted in thicker fibers with an average diameter of around 0.47 μm. At the highest concentration tested, 17%, the fibers reached an average diameter of 0.71 μm. This trend suggests that higher PVC concentrations lead to increased fiber thickness during electrospinning.

**Figure 5:**
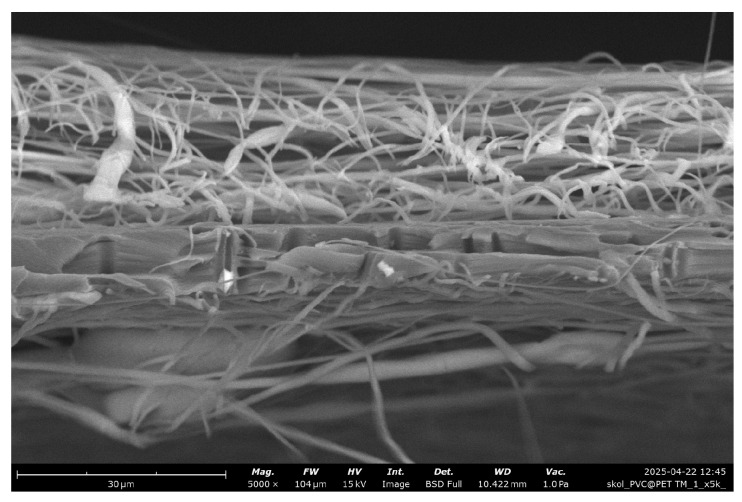
Cross-section SEM microphotographs of PET TeMs covered with PVC fibers (15%).

Figure 5 presents a cross-sectional view of the membrane obtained by electrospinning a 15% PVC solution. The image clearly illustrates a layer of randomly oriented PVC nanofibers deposited on the surface of the PET substrate. The fibrous mat exhibits a typical non-woven structure, with fibers forming an interconnected porous network. Between the electrospun layers, the PET TeMs structure with vertically aligned cylindrical pores is visible.

Elemental analysis of PVC fibers on the surface of PET TeMs made by SEM EDX showed the following results: atomic content (C) = 87.4%, (Cl) = 8.6%, (O) = 4.1%. This composition is not the same as that of PVC, so there is some residual effect coming from the PET layer. The mechanical properties of the membranes were also evaluated, and they did not differ from the original PET TeMs, and were equal to 260 kPa for membranes with a pore diameter of 1727 nm, 281 kPa for 1159 nm, and 213 kPa for 2494 nm.

**Figure 6:**
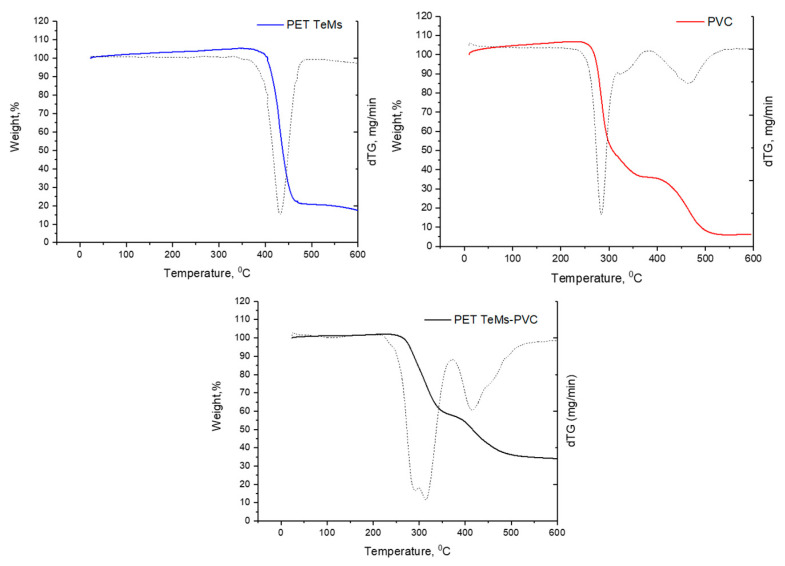
TGA curves of PET TeMs (blue), pure PVC (red), and PET TeMs-PVC (black), dashed lines correspond to the dTG curves of the respective samples.

TGA curves of PET TeMs, pure PVC, and PET TeMs-PVC are presented in Figure 6. TGA curve of PET TeMs is characterized by one main peak at 432 °C, and weight loss was 84%. Dehydrochlorination of PVC occurred at 210–350 °C. Further weight loss is associated with cyclization of conjugated polyene chains to form aromatic compounds in the temperature range 350–500 °C [67]. TGA curve of PET TeMs-PVC is also characterized by two main peaks of mass loss at 299 °C (43.6%) and at 417 °C (23%). Also, it should be noted that the slight weight increase observed between room temperature and 200 °C is not attributed to gas adsorption on the membrane surface. Instead, this phenomenon is most likely caused by buoyancy-related artifacts inherent to TGA. During the initial heating phase, the aerodynamic drag generated by the gas flow within the furnace can result in an apparent mass gain. This effect has been discussed in the literature and is considered a common source of experimental deviation in TGA measurements [68,69]. This interpretation is further supported by the absence of any corresponding changes in heat flow, as no thermal events were detected in the DTA signal.

The interfacial adhesion between electrospun PVC nanofiber layers and PET TeMs was evaluated using progressive load scratch testing. The resulting average F_cr_ is around 1.1 N, and δ_adh_ is 110 ± 6 kPa. These values indicate a moderate level of physical adhesion between the electrospun PVC nanofibers and the PET TeMs. Such adhesion is sufficient to ensure mechanical stability under mild operating conditions, but may not withstand more aggressive environments without additional surface treatment or binding agents. Thus, in membrane-based water treatment processes such as MD or filtration, these types of membranes are typically recommended for use under operating pressures not exceeding 1 bar. In MD, the typical transmembrane pressure can be 1–2 bar (but does not usually exceed 1 bar). The observed adhesion strength is of the same order of magnitude, suggesting that the electrospun PVC fibers are unlikely to delaminate or detach under such conditions. Therefore, the PET TeMs–PVC exhibit adequate adhesion performance for use in water purification by MD.

### 3.2. Testing of Hybrid Membranes in the Separation of Water–Oil Mixtures

Hydrophobic properties of hybrid PET TeMs-PVC membranes (with 15% PVC) make them suitable for use in the separation of water–oil emulsions by filtration and in the MD process. The data obtained on the separation of the cetane–water emulsion is shown in Table 1. The corresponding time of passage of cetane at a pressure of 900, 800, 700 mbar for PET TeMs coated with PVC (15%). The degree of purification was 99–99.5%.

**Figure 7:**
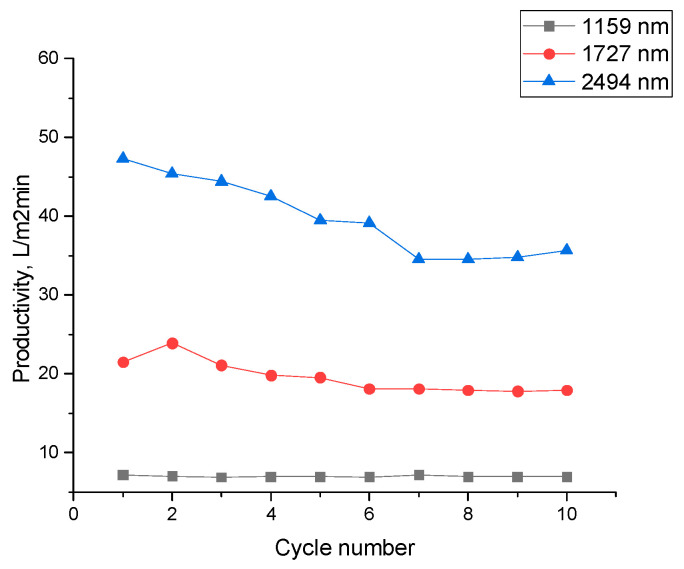
Productivity of a hybrid PET TeMs-PVC membrane versus number of cycles with different pore diameters at a pressure of 700 mbar for the separation of a cetane–water emulsion.

An increase in membrane pore size and applied pressure leads to higher productivity while maintaining stable separation efficiency. As a result, the developed membranes demonstrate effective performance in the separation of water–oil emulsions. The dependence of the performance of the separation of water–oil emulsions on pore diameter in 10 cycles is shown in Figure 7. It should be noted that the efficiency decreases with the cycle, but only slightly for membranes with 1159 nm pores (3.1%), while for 1727 nm and 2494 nm membranes, the decrease was 16.7% and 24.6%, respectively. This reduction in flux can be attributed to the fact that membranes with larger pores allow a greater volume of emulsion to pass through per unit time, which increases the exposure of pore walls to oil droplets. This facilitates more rapid fouling and partial pore wetting.

Flux recovery was calculated after 10 filtration cycles using the method described in [70]. Following membrane cleaning, the flux recovery was 96% for PET TeMs-PVC with 1159 nm pores, 94% for 1727 nm pores, and 91% for 2494 nm pores.

Several studies have investigated membrane technologies for separating oil–water emulsions, addressing aspects such as selectivity, fouling resistance, and productivity. For instance, Zhang et al. utilized polycarbonate (PC) TeMs modified with PDDA/PSS polyelectrolyte multilayers to treat emulsions stabilized by surfactants, including cetyltrimethylammonium bromide (CTAB) and sodium dodecyl sulfate (SDS). While the separation degree exceeded 99.98%, the flux was relatively low (24 L·m^−2^·h^−1^), limiting their industrial applicability despite excellent rejection performance [71]. Banat et al. developed a superhydrophobic PVDF membrane designed for water–oil emulsions (n-heptane–water system) using Span-80 as an emulsifier. Their membrane demonstrated a separation efficiency above 95%, with a moderate flux around 150 L·m^−2^·h^−1^, though a decline was observed over long-term operation [72]. Yang et al. used a porous poly(phenylene sulfide) (PPS) membrane for separating both surfactant-free and stabilized water–oil emulsions, achieving a separation degree of over 99.7%, with water content in the permeate reduced to below 300 ppm [73]. A more recent study by Wu et al. introduced a smart wood-based membrane functionalized with photoswitchable polymers for surfactant-stabilized diesel–water and oil–water emulsions. Their system demonstrated both ultra-high separation efficiency (>99.99%) and exceptional fluxes up to 4392 L·m^−2^·h^−1^ [74]. In comparison, PET TeMs-PVC composite membranes developed in our study were specifically tested on cetane–water emulsions. The membrane with the largest pore size (2494 ± 81 nm) achieved a maximum flux of 47.3 L·m⁻^2^·min⁻^1^ (2838 L·m^−2^·h^−1^) at 700 mbar vacuum pressure.

### 3.3. Testing of Hybrid Membranes in Membrane Distillation

PET TeMs-PVC membranes containing 15% PVC and different pore sizes of 1159 ± 27 nm, 1727 ± 73 nm, and 2494 ± 81 nm were evaluated in DCMD for the treatment of model saline solutions, river water, and low-level liquid radioactive waste (LLLRW). The design and configuration of the experimental setup are detailed in our earlier publication [75]. The influence of membrane pore size and sodium chloride concentration on water flux and salt rejection was investigated, with the corresponding results summarized in Table 2.

Results show a decrease in water flux with increasing salt concentration. It can be due to the fact that salt concentration influences the feed solution’s vapor pressure. Water flux for the solution of NaCl with a concentration of 7.5 g/L is 16.60 kg/m^2^·h (for membranes with pore diameters of 2494 ± 81 nm), and is 12.51 kg/m^2^·h for 30 g/L. The degree of rejection is mainly dependent on pore diameter. Increasing the pore diameter from 1159 nm to 2494 nm led to a slight decrease in salt rejection from 98.67 to 95.64% (for 30 g/L NaCl).

When benchmarked against commercial PTFE membranes, such as those studied by Zhang et al., similar trends were observed: PTFE membranes with larger pores achieved higher fluxes (up to ~26 kg/m^2^·h) [76]. In a more recent study, Hegde C. et al. demonstrated that hybrid PTFE-based membranes achieved salt rejection of 99% and productivity around 2726 kg/m^2^·h in direct contact MD with high energy efficiency (up to 67.3%), further supporting the relevance of pore design and material composition in MD membrane performance [77]. Additionally, Liu et al. emphasized the importance of addressing concentration polarization and fouling, both of which may affect membrane performance, particularly under long-term operation or higher salinity conditions [78]. Gonzalez et al. presented insights into sustainable and energy-efficient MD configurations using PTFE/PVDF membranes, achieving >99% rejection and improved performance through system-level innovations [79]. Furthermore, Arslan et al. demonstrated that nanoparticle-enhanced PVDF membranes achieved superior heat and mass transfer efficiency (up to 2.0 L/m^2^·h) and resistance to wetting and fouling, suggesting potential for further performance optimization in MD systems [80]. Taken together, the PET TeMs-PVC membranes evaluated here demonstrate competitive performance relative to commercial and advanced membranes.

High water fluxes while maintaining a degree of purification make these types of membranes quite promising for practical use. Testing of real solutions was carried out by using LLLRW samples obtained from the Research Nuclear Reactor WWR-K (Almaty, Kazakhstan) and river water. Water from rivers was collected and purified without any pretreatment. There is a slight decrease in water fluxes in comparison with model NaCl solutions, probably due to the multicomponent composition of water from the river, including biological pollutants that can lead to membrane fouling. The elemental composition of water before and after purification was studied using the method of ICP-OES. Results are presented in Table 3. For all elements, a sharp decrease in concentrations is observed after water treatment, with a high degree of purification. Cation concentrations are lower than the maximum permissible concentrations for drinking water.

The next stage of the research was the treatment of water that contains radioisotopes. In our previous studies [19,20] on TeMs with small pore diameters, despite the high degrees of purification achieved, the performance of the membranes needed to be improved. Therefore, the membranes obtained in this work may be of greater practical importance from this point of view. LLLRW contains ions and radioisotopes presented in Table 4 and Table 5.

Activity of radioisotopes ^124^Sb, ^65^Zn, ^60^Co, ^57^Co, ^137^Cs, and ^134^Cs before and after treatment was monitored by a Gamma Ray Spectrometer. It is seen from Table 5 that PET TeMs-PVC with pore diameters of 1159 ± 27 nm and 2494 ± 81 nm can clean water from radioisotopes with a high degree of purification. According to the hygienic standards for ensuring radiation safety in the Republic of Kazakhstan, purified water does not exceed the content of radioisotopes for drinking water (data presented in Table 5). However, the use of PET TeMs-PVC with a pore diameter of 2494 ± 81 nm leads to an excess of ^60^Co content.

Comparing the results obtained on the use of a PET-TeMs covered with PVC fibers in MD with those previously published using nanofiber membranes [81] and TeMs [19,71,75], it can be argued that hybrid membranes are significantly superior to hydrophobic TeMs in water fluxes since the hybrid structure of the membrane allows us to significantly increase the pore size of TeMs. Specifically, the PET-TeMs-PVC membranes demonstrated water fluxes of up to 16.6 kg/m^2^·h and salt rejection reaching 99.1% at a NaCl concentration of 15 g/L. In contrast, conventional hydrophobized TeMs typically show lower water fluxes in the range of 5–10 kg/m^2^·h under similar conditions, with slightly reduced salt rejection values around 95–98% [19,71,75]. Electrospun nanofiber membranes made of different polymers and structures achieve water fluxes from 1.83 to 78 kg/m^2^·h with a salt rejection degree ranging around 99.9% [81]. Thus, the incorporation of nanofiber layers and the development of hybrid TeMs markedly improve membrane performance relative to conventional hydrophobized TeMs.

## 4. Conclusions

In this study, hybrid membranes were successfully fabricated by electrospinning PVC fibers onto PET TeMs, resulting in a significant improvement in hydrophobicity (contact angle up to 155°) and the enhancement of membrane performance in both membrane distillation and oil–water separation. The hybrid membranes demonstrated high water fluxes (up to 17.3 kg/m^2^·h for MD and 47.3 L/m^2^·min for oil–water separation) while maintaining excellent rejection rates (>98%). The integration of electrospun PVC nanofibers on both sides of TeMs allowed us to overcome the common limitation of low permeability associated with TeMs without compromising selectivity.

The results clearly demonstrate how these hybrid membranes offer a promising route for addressing the challenges of treating complex liquid mixtures, including LLLRW and water–oil emulsions. Importantly, the membranes showed stable operation during multi-cycle tests and effectively removed both radionuclides and conventional inorganic contaminants. However, several limitations remain. Despite the promising fluxes, long-term performance under real industrial conditions, including membrane fouling, mechanical integrity under prolonged use, and cleaning efficiency, require further investigation. Additionally, scale-up and module integration for full-scale deployment have not yet been addressed. Future research will focus on further tuning the fiber morphology and membrane architecture to enhance antifouling and anti-wetting properties, exploring other polymer–fiber combinations, and integrating functional additives (e.g., nanoparticles) to impart additional selectivity or photocatalytic self-cleaning properties.

## Figures and Tables

**Table 1:** Separation of cetane–water emulsion by hybrid PET TeMs-PVC membrane.

Membrane	Vacuum Pressure, mbar	Productivity, L/m^2^·min
PET TeMs-PVC with pore size of 1159 ± 27 nm	900	4.4
	800	5.8
	700	7.0
PET TeMs-PVC with pore size of 1727 ± 73 nm	900	4.6
	800	14.6
	700	21.5
PET TeMs-PVC with pore size of 2494 ± 81 nm	900	15.2
	800	35.4
	700	47.3

**Table 2:** Membrane distillation of saline solutions using PET TeMs-PVC membrane.

	PET TeMs-PVC with Pore Diameter of 1159 ± 27 nm		PET TeMs-PVC with Pore Diameter of 1727 ± 73 nm		PET TeMs-PVC with Pore Diameter of 2494 ± 81 nm	
	Degree of Salt Rejection, %	Water Flow, kg/m^2^·h	Degree of Salt Rejection, %	Water Flow, kg/m^2^·h	Degree of Salt Rejection, %	Water Flow, kg/m^2^·h
NaCl 7.5 g/L	97.2	10.14	96.4	14.96	95.5	16.60
NaCl 15 g/L	98.7	9.74	99.1	13.36	97.15	13.61
NaCl 30 g/L	98.67	6.69	97.12	11.85	95.64	12.51

**Table 3:** Results of river water purification by MD with hybrid PET TeMs-PVC.

	Concentration in the Feed, µg/L	PET TeMs-PVC (1159 ± 27 nm), 2.5 h of Operation, µg/L	PET TeMs-PVC (1159 ± 27 nm), 5 h of Operation, µg/L	PET TeMs-PVC (1727 ± 73 nm), 2.5 h of Operation, µg/L	PET TeMs-PVC (1727 ± 73 nm), 5 h of Operation, µg/L	PET TeMs-PVC (2494 ± 81 nm), 2.5 h of Operation, µg/L	PET TeMs-PVC (2494 ± 81 nm), 5 h of Operation, µg/L
Water Flux, kg/m^2^·h	-	6.42 kg/m^2^·h		11.5 kg/m^2^·h		12.2 kg/m^2^·h	
Al (σ = ±32%)	<30	<3	<3	<3	<3	<3	<3
Fe (σ = ±10%)	3.23	<0.5	<0.5	<0.5	<0.5	<0.5	<0.5
Mn (σ = ±32%)	<4	<0.4	<0.4	<0.4	<0.4	3.3	3.1
Sr (σ = ±15%)	1240	<0.5	<0.5	3.0	4.9	23.1	28.2
Ca (σ = ±16%)	152,000	130	80	32	54	267	333
Mg (σ = ±15%)	43,000	23	27	110	170	780	950
Na (σ = ±15%)	235,000	1870	1888	1070	1710	5110	7220

**Table 4:** Results of LLRW purification by MD with hybrid PET TeMs-PVC.

	Concentration in the Feed, µg/L	PET TeMs-PVC (1159 ± 27 nm), 2.5 h of Operation, µg/L	PET TeMs-PVC (1159 ± 27 nm), 5 h of Operation, µg/L	PET TeMs-PVC (1727 ± 73 nm), 2.5 h of Operation, µg/L	PET TeMs-PVC (1727 ± 73 nm), 5 h of Operation, µg/L	PET TeMs-PVC (2494 ± 81 nm), 2.5 h of Operation, µg/L	PET TeMs-PVC (2494 ± 81 nm), 5 h of Operation, µg/L
Water Flux, kg/m^2^·h	-	14.9 kg/m^2^·h		15.2 kg/m^2^·h		17.3 kg/m^2^·h	
Al (σ = ±32%)	104	<3	<3	<3	<3	<3	<3
Co (σ = ±20%)	1.5	<0.1	<0.1	<0.1	<0.1	<0.1	<0.1
Cs (σ = ±20%)	0.20	<0.05	<0.05	<0.05	<0.05	<0.05	<0.05
Fe (σ = ±10%)	6800	<0.5	5.3	<0.5	10.8	68.5	130
Mn (σ = ±32%)	4.4	<0.4	<0.4	<0.4	<0.4	<0.4	<0.4
Mo (σ = ±26%)	970	<0.4	0.87	<0.4	5.4	15.9	46.4
Sb (σ = ±26%)	30	<0.3	<0.3	<0.3	<0.3	0.58	1.4
Sr (σ = ±15%)	270	<0.5	<0.5	<0.5	<0.5	2.2	2.7
Ca (σ = ±16%)	21,300	<20	<20	<20	<20	74	150
Mg (σ = ±15%)	990	<10	<10	<10	<10	54	110
Na (σ = ±15%)	440,000	250	450	230	2630	9920	23,500

**Table 5:** Radioisotope composition of feed waste solution and permeate solution after the DCMD process.

	^134^Cs	^137^Cs	^60^Co	^57^Co	^124^Sb	^65^Zn
Activity of the feed, Bq/kg	77.0 ± 7.5	451 ± 45	2052 ± 200	32,882 ± 3200	1385 ± 135	4421 ± 440
PET TeMs-PVC (1159 ± 27 nm,) 5 h of operation, Bq/kg	<1.3	<1.1	<1.2	16.0 ± 1.5	<11	<3.2
PET TeMs-PVC (1727 ± 73 nm), 5 h of operation, Bq/kg	2.6 ± 1.0	2.33 ± 0.95	<1.3	172 ± 15	<12	<3.7
PET TeMs-PVC (2494 ± 81 nm), 5 h of operation, Bq/kg	3.9 ± 1.8	16.7 ± 2.2	92.9 ± 9.0	1448 ± 140	34 ± 15	<6.7
Intervention levels (Bq/kg) for the content of individual radionuclides in drinking water, Bq/kg *	7.2	11	40	650	55	35

*—On approval of hygienic standards for ensuring radiation safety (Republic of Kazakhstan).

## Data Availability

The original contributions presented in this study are included in the article. Further inquiries can be directed at the corresponding author.

## References

[1] Bin Abid M., Wahab R.A., Salam M.A., Gzara L., Moujdin I.A. (2023). Desalination Technologies, Membrane Distillation, and Electrospinning, an Overview. Heliyon.

[2] Zhang X., Koirala R., Pramanik B., Fan L., Date A., Jegatheesan V. (2023). Challenges and Advancements in Membrane Distillation Crystallization for Industrial Applications. Environ. Res..

[3] Ngo M.T.T., Nguyen H.N.M., Nguyen N.C., Nguyen P.T., Bui X.T. (2023). Chapter 14—Applications and Challenges of Membrane Distillation in Water Reuse. Current Developments in Biotechnology and Bioengineering: Membrane Technology for Sustainable Water and Energy Management.

[4] Julian H., Gusnawan P.J., Wonoputri V., Setiadi T. (2023). Chapter 16—Membrane Distillation for Wastewater Treatment: Recent Advances in Process Optimization and Membrane Modification. Current Developments in Biotechnology and Bioengineering: Membrane Technology for Sustainable Water and Energy Management.

[5] Kalla S., Piash K.P.S., Sanyal O. (2022). Anti-Fouling and Anti-Wetting Membranes for Membrane Distillation. J. Water Process Eng..

[6] Chen X., Chen T., Li J., Qiu M., Fu K., Cui Z., Fan Y., Drioli E. (2019). Ceramic Nanofiltration and Membrane Distillation Hybrid Membrane Processes for the Purification and Recycling of Boric Acid from Simulative Radioactive Waste Water. J. Membr. Sci..

[7] Hu X., Guo J., An A.K.J., Chopra S.S. (2023). Electrospun Nanofibrous Membranes for Membrane Distillation Application—A Dynamic Life Cycle Assessment (DLCA) Approach. Water Res..

[8] Julian H., Nurgirisia N., Qiu G., Ting Y.P., Wenten I.G. (2022). Membrane Distillation for Wastewater Treatment: Current Trends, Challenges and Prospects of Dense Membrane Distillation. J. Water Process Eng..

[9] Qoriati D., Susane H., Lin J.L., Wang Y.F., You S.J. (2023). Chapter 15—Membrane Distillation Technology Applied in Water Resources. Current Developments in Biotechnology and Bioengineering: Membrane Technology for Sustainable Water and Energy Management.

[10] Wen X., Li F., Zhao X. (2016). Removal of Nuclides and Boron from Highly Saline Radioactive Wastewater by Direct Contact Membrane Distillation. Desalination.

[11] Prado E.S.P., Miranda F.S., Araujo L.G., Petraconi G., Baldan M.R., Essiptchouk A., Potiens A.J. (2021). Experimental Study on Treatment of Simulated Radioactive Waste by Thermal Plasma: Temporal Evaluation of Stable Co and Cs. Ann. Nucl. Energy.

[12] Du X., Tong W., Zhou X., Luo J., Liu Y., Wang Y., Li P., Zhang Y. (2024). An Efficient Approach for the Treatment of Radioactive Waste Perfluoropolyether Lubricants via a Synergistic Effect of Thermal Catalysis and Immobilization. J. Environ. Sci..

[13] Barlow S.T., Fisher A.J., Bailey D.J., Blackburn L.R., Stennett M.C., Hand R.J., Morgan S.P., Hyatt N.C., Corkhill C.L. (2021). Thermal Treatment of Nuclear Fuel-Containing Magnox Sludge Radioactive Waste. J. Nucl. Mater..

[14] Singh B.K., Um W. (2023). Application of Clay Materials for Sorption of Radionuclides from Waste Solutions. Minerals.

[15] Metwally S.S., Borai E.H., Hamed M.M., Mohamed T.M., Hamed M.G., Mohamed W.R. (2023). Low-Cost and Eco-Friendly Biosorbent for Effective Removal of Hazardous Ions from Simulated Radioactive Liquid Waste. Arab. J. Chem..

[16] Osmanlioglu A.E. (2006). Treatment of Radioactive Liquid Waste by Sorption on Natural Zeolite in Turkey. J. Hazard. Mater..

[17] Yu S., Wei J., Zhang X., Zhao X. (2024). Removal of Silver Colloids from Radioactive Wastewater by Means of Ultrafiltration. Radiat. Phys. Chem..

[18] Wei Y., Cao L., Zhu J., Wang L., Yao H., Sun H., Xia X., Zhao H., Ji T., Ni S. (2024). Radioactive Strontium Ions Sieving through Reduced Graphene Oxide Membrane. J. Membr. Sci..

[19] Korolkov I.V., Yeszhanov A.B., Zdorovets M.V., Gorin Y.G., Güven O., Dosmagambetova S.S., Khlebnikov N.A., Serkov K.V., Krasnopyorova M.V., Milts O.S. (2019). Modification of PET Ion Track Membranes for Membrane Distillation of Low-Level Liquid Radioactive Wastes and Salt Solutions. Sep. Purif. Technol..

[20] Zdorovets M.V., Yeszhanov A.B., Korolkov I.V., Güven O., Dosmagambetova S.S., Shlimas D.I., Zhatkanbayeva Z.K., Zhidkov I.S., Kharkin P.V., Gluchshenko V.N. (2020). Liquid Low-Level Radioactive Wastes Treatment by Using Hydrophobized Track-Etched Membranes. Prog. Nucl. Energy.

[21] Yu S., Zhang X., Li F., Zhao X. (2018). Poly(Vinyl Pyrrolidone) Modified Poly(Vinylidene Fluoride) Ultrafiltration Membrane via a Two-Step Surface Grafting for Radioactive Wastewater Treatment. Sep. Purif. Technol..

[22] Zhang X., Niu L., Li F., Zhao X., Hu H. (2017). Enhanced Rejection of Cations by Low-Level Cationic Surfactant during Ultrafiltration of Low-Level Radioactive Wastewater. Sep. Purif. Technol..

[23] Weerasekara N.A., Choo K.H., Choi S.J. (2013). Metal Oxide Enhanced Microfiltration for the Selective Removal of Co and Sr Ions from Nuclear Laundry Wastewater. J. Membr. Sci..

[24] Chung Y., Park D., Kim H., Kim Y., Kang S. (2021). The Impact of Gamma-Irradiation from Radioactive Liquid Wastewater on Polymeric Structures of Nanofiltration (NF) Membranes. J. Hazard. Mater..

[25] Zhang Y., Yang Y., Takizawa S., Kuroda K., Jia Z., Liu P., Wei H., Graham N.J.D. (2024). Development of a Composite MoS2/PEI Nanofiltration Membrane for Radionuclides Efficient Removal from Aquatic Environments. Sep. Purif. Technol..

[26] Wang C., Wang Y., Qin H., Lin H., Chhuon K. (2020). Application of Microfiltration Membrane Technology in Water Treatment. IOP Conf. Ser. Earth Environ. Sci..

[27] Lee S., Kim Y., Park J., Shon H.K., Hong S. (2018). Treatment of Medical Radioactive Liquid Waste Using Forward Osmosis (FO) Membrane Process. J. Membr. Sci..

[28] Lee B.S. (2015). Nuclide Separation Modeling through Reverse Osmosis Membranes in Radioactive Liquid Waste. Nucl. Eng. Technol..

[29] Chen B., Yu S., Zhao X. (2021). The Separation of Radionuclides and Silicon from Boron-Containing Radioactive Wastewater with Modified Reverse Osmosis Membranes. Process Saf. Environ. Prot..

[30] Chen D., Zhao X., Li F., Zhang X. (2016). Rejection of Nuclides and Silicon from Boron-Containing Radioactive Waste Water Using Reverse Osmosis. Sep. Purif. Technol..

[31] Jia X., Lan L., Wang T., Wang Y., Ye C. (2024). The Impact of Gamma-Irradiation on Physical Properties and Membrane Distillation Performance of PTFE and PVDF Hollow Fiber Membrane. Ann. Nucl. Energy.

[32] Jiang M., Fang Z., Liu Z., Huang X., Wei H., Yu C.Y. (2023). Application of Membrane Distillation for Purification of Radioactive Liquid. Clean. Eng. Technol..

[33] Li S., He Z., Xiao D., Guo L. (2022). Study on the Treatment of Radioactive Wastewater by Non-Contact Membrane Distillation. Sep. Purif. Technol..

[34] Nie X., Hu X., Liu C., Xia X., Dong F. (2021). Decontamination of Uranium Contained Low-Level Radioactive Wastewater from UO_2_ Fuel Element Industry with Vacuum Membrane Distillation. Desalination.

[35] Yeszhanov A.B., Korolkov I.V., Kh Shakayeva A., Lissovskaya L.I., Zdorovets M.V. (2023). Preparation of Poly(Ethylene Terephthalate) Track-Etched Membranes for the Separation of Water-Oil Emulsions. Eurasian J. Chem..

[36] Liu J., Wang Q., Shan H., Guo H., Li B. (2019). Surface Hydrophobicity Based Heat and Mass Transfer Mechanism in Membrane Distillation. J. Membr. Sci..

[37] Yadav A., Labhasetwar P.K., Shahi V.K. (2022). Membrane Distillation Crystallization Technology for Zero Liquid Discharge and Resource Recovery: Opportunities, Challenges and Futuristic Perspectives. Sci. Total Environ..

[38] Jawed A.S., Nassar L., Hegab H.M., van der Merwe R., Al Marzooqi F., Banat F., Hasan S.W. (2024). Recent Developments in Solar-Powered Membrane Distillation for Sustainable Desalination. Heliyon.

[39] Zhang R., Chen Y., Wang H., Duan X., Ren Y. (2024). A Janus Membrane with Silica Nanoparticles Interlayer for Treating Coal Mine Water via Membrane Distillation. Sep. Purif. Technol..

[40] Zhao J., Wang S., Zhao S., Chen L., Meng F., Tian X. (2024). A Non-Fluorinated Liquid-like Membrane with Excellent Anti-Scaling Performance for Membrane Distillation. Chin. Chem. Lett..

[41] Hou X., Wang J., Zhang R., Gao Y., Zhou Z., Li H., Lu Y., Tang L. (2024). Preparation of PVDF-NSiO_2_/PVSQ High-Flux Composite Membrane by Chemical Grafting and Separation of Composite Brine by Membrane Distillation. J. Environ. Chem. Eng..

[42] Zhang Y., Guo F. (2024). Mitigating Near-Surface Polarizations in Membrane Distillation via Membrane Surface Decoration. Desalination.

[43] Jia F., Li J., Wang J., Sun Y. (2017). Removal of Strontium Ions from Simulated Radioactive Wastewater by Vacuum Membrane Distillation. Ann. Nucl. Energy.

[44] Jia F., Li J., Wang J. (2017). Recovery of Boric Acid from the Simulated Radioactive Wastewater by Vacuum Membrane Distillation Crystallization. Ann. Nucl. Energy.

[45] Barsbay M., Güven O. (2014). Grafting in Confined Spaces: Functionalization of Nanochannels of Track-Etched Membranes. Radiat. Phys. Chem..

[46] Korolkov I.V., Mashentseva A.A., Güven O., Gorin Y.G., Zdorovets M.V. (2018). Protein Fouling of Modified Microporous PET Track-Etched Membranes. Radiat. Phys. Chem..

[47] Zhumanazar N., Korolkov I.V., Yeszhanov A.B., Shlimas D.I., Zdorovets M.V. (2023). Electrochemical Detection of Lead and Cadmium Ions in Water by Sensors Based on Modified Track-Etched Membranes. Sens. Actuators A Phys..

[48] Shakayeva A.K., Munasbaeva K.K., Zhumazhanova A.T., Zdorovets M.V., Korolkov I.V. (2023). Electrochemical Sensors Based on Modified Track–Etched Membrane for Non-Enzymatic Glucose Determination. Microchem. J..

[49] Parmanbek N., Sütekin D.S., Barsbay M., Mashentseva A.A., Zheltov D.A., Aimanova N.A., Jakupova Z.Y., Zdorovets M.V. (2022). Hybrid PET Track-Etched Membranes Grafted by Well-Defined Poly(2-(dimethylamino)ethyl methacrylate) Brushes and Loaded with Silver Nanoparticles for the Removal of As(III). Polymers.

[50] Mashentseva A.A. (2019). Effect of the Oxidative Modification and Activation of Templates Based on Poly(Ethylene Terephthalate) Track-Etched Membranes on the Electroless Deposition of Copper and the Catalytic Properties of Composite Membranes. Pet. Chem..

[51] Mashentseva A.A., Barsbay M., Aimanova N.A., Zdorovets M.V. (2021). Application of Silver-Loaded Composite Track-Etched Membranes for Photocatalytic Decomposition of Methylene Blue under Visible Light. Membranes.

[52] Korolkov I.V., Kuandykova A., Yeszhanov A.B., Güven O., Gorin Y.G., Zdorovets M.V. (2020). Modification of Pet Ion-Track Membranes by Silica Nanoparticles for Direct Contact Membrane Distillation of Salt Solutions. Membranes.

[53] Korolkov I.V., Yeszhanov A.B., Gorin Y.G., Zdorovets M.V., Khlebnikov N.A., Serkov K.V. (2018). Hydrophobization of PET Track-Etched Membranes for Direct Contact Membrane Distillation. Mater. Res. Exp..

[54] Shakayeva A.K., Yeszhanov A.B., Zhumazhanova A.T., Korolkov I.V., Zdorovets M.V. (2024). Fabrication of Hydrophobic PET Track-Etched Membranes Using 2,2,3,3,4,4,4-Heptafluorobutyl Methacrylate for Water Desalination by Membrane Distillation. Eurasian J. Chem..

[55] Sangeetha V., Kaleekkal N.J. (2022). Investigation of the Performance of Dual-Layered Omniphobic Electrospun Nanofibrous Membranes for Direct Contact Membrane Distillation. J. Environ. Chem. Eng..

[56] Kong Z., Hu Y., Yan M., Jiang N., Meng L., Huang M. (2024). Novel Dual-Layer ZIF-71/PH-PSF Electrospun Nanofiber for Robust Membrane Distillation. Sep. Purif. Technol..

[57] Xu Y., Yu Y., Song C., Zhu Y., Song C., Fan X., You Z. (2022). One-Step Preparation of Efficient SiO_2_/PVDF Membrane by Sol-Gel Strategy for Oil/Water Separation under Harsh Environments. Polymer.

[58] Oh Y., Kim J., Lee E., Lee J., Jeong S. (2023). Effect of Fabrication Technique on Membrane Distillation Performance of Photothermal Membrane: Electrospinning vs Electrospraying. Desalination.

[59] Jiang S., Liang S., Hu C., Fan Y., Su Z., Geng Z., Wang C. (2024). High Performance Bimetallic ZIF-CoZn@PVDF-HFP Composite Nanofiber Membrane for Membrane Distillation Based on Coaxial Electrospinning Technology. Sep. Purif. Technol..

[60] Muslimova I.B., Zhatkanbayeva Z.K., Omertasov D.D., Melnikova G.B., Yeszhanov A.B., Güven O., Chizhik S.A., Zdorovets M.V., Korolkov I.V. (2023). Stimuli-Responsive Track-Etched Membranes for Separation of Water—Oil Emulsions. Membranes.

[61] Yeszhanov A.B., Korolkov I.V., Güven O., Melnikova G.B., Dosmagambetova S.S., Borissenko A.N., Nurkassimov A.K., Kassymzhanov M.T., Zdorovets M.V. (2024). Effect of Hydrophobized PET TeMs Membrane Pore-Size on Saline Water Treatment by Direct Contact Membrane Distillation. RSC Adv..

[62] Yeszhanov A.B., Korolkov I.V., Dosmagambetova S.S., Zdorovets M.V., Güven O. (2021). Recent Progress in the Membrane Distillation and Impact of Track-Etched Membranes. Polymers.

[63] Pham L.Q., Uspenskaya M.V., Olekhnovich R.O., Bernal R.A.O. (2021). A Review on Electrospun Pvc Nanofibers: Fabrication, Properties, and Application. Fibers.

[64] Asmatulu R., Muppalla H., Veisi Z., Khan W.S., Asaduzzaman A., Nuraje N. (2013). Study of Hydrophilic Electrospun Nanofiber Membranes for Filtration of Micro and Nanosize Suspended Particles. Membranes.

[65] Huang Z.M., Zhang Y.Z., Kotaki M., Ramakrishna S. (2003). A Review on Polymer Nanofibers by Electrospinning and Their Applications in Nanocomposites. Compos. Sci. Technol..

[66] El Messiry M., Fadel N. (2019). Study of Poly(Vinyl Chloride) Nanofiber Structured Assemblies as Oil Sorbents. J. Text. Inst..

[67] Jia P., Wang R., Hu L., Zhang M., Zhou Y. (2017). Self-Plasticization of PVC via Click Reaction of a Monooctyl Phthalate Derivative. Pol. J. Chem. Technol..

[68] Crewe R.J., Staggs J.E.J., Williams P.T. (2007). Drag-Induced Apparent Mass Gain in Thermogravimetry. Polym. Degrad. Stab..

[69] Tsioptsias C. (2022). On the Latent Limit of Detection of Thermogravimetric Analysis. Measurement.

[70] Muslimova I.B., Zhumanazar N., Melnikova G.B., Yeszhanov A.B., Zhatkanbayeva Z.K., Chizhik S.A., Zdorovets M.V., Güven O., Korolkov I.V. (2024). Preparation and Application of Stimuli-Responsive PET TeMs: RAFT Graft Block Copolymerisation of Styrene and Acrylic Acid for the Separation of Water–Oil Emulsions. RSC Adv..

[71] Zhang G., Li L., Huang Y., Hozumi A., Sonoda T., Su Z. (2018). Fouling-Resistant Membranes for Separation of Oil-in-Water Emulsions. RSC Adv..

[72] Hai A., Selvaraj M., Govindan B., Krishnamoorthy R., Hasan S.W., Banat F. (2020). Demulsification Performance of Superhydrophobic PVDF Membrane: A Parametric Study. J. Membr. Sci. Res..

[73] Yang C., Han N., Wang W., Zhang W., Han C., Cui Z., Zhang X. (2018). Fabrication of a PPS Microporous Membrane for Efficient Water-in-Oil Emulsion Separation. Langmuir.

[74] Wu J., Cui Z., Yu Y., Han H., Tian D., Hu J., Qu J., Cai Y., Luo J., Li J. (2023). A 3D Smart Wood Membrane with High Flux and Efficiency for Separation of Stabilized Oil/Water Emulsions. J. Hazard. Mater..

[75] Korolkov I.V., Gorin Y.G., Yeszhanov A.B., Kozlovskiy A.L., Zdorovets M.V. (2018). Preparation of PET Track-Etched Membranes for Membrane Distillation by Photo-Induced Graft Polymerization. Mater. Chem. Phys..

[76] Zhang J., Duke M., Ostarcevic E., Dow N., Gray S., Li J. (2009). Performance of New Generation Membrane Distillation Membranes. Water Supply.

[77] Hegde C., Ribeiro R. (2022). Preparation and Characterization of Hydrophobic Membranes and Their Seawater Desalination Performance Study by Direct Contact Membrane Distillation. Nat. Environ. Pollut. Technol..

[78] Liu C., Liu G., Sun W., Ren X., Qiu L., Zhang S., Xie K., Zuo C. (2022). Performance Enhancement of Membrane Distillation in the Desalina-Tion of Sea Water and Brackish Water. Proceedings of the 2022 International Conference on Optoelectronic Information and Functional Materials (OIFM 2022).

[79] González D., Amigo J., Suárez F. (2017). Membrane Distillation: Perspectives for Sustainable and Improved Desalination. Renew. Sustain. Energy Rev..

[80] Arslan M., Haizhen X., Khalid Khan M.N., Ali K., Tariq Baig Z. (2024). Bin Innovative Materials for Enhanced Heat and Mass Transfer in Membrane Distillation. Proceedings of the 2024 Horizons of Information Technology and Engineering (HITE).

[81] Pan C.Y., Xu G.R., Xu K., Zhao H.L., Wu Y.Q., Su H.C., Xu J.M., Das R. (2019). Electrospun Nanofibrous Membranes in Membrane Distillation: Recent Developments and Future Perspectives. Sep. Purif. Technol..

